# A novel treatment for the fixation of displaced femoral neck fractures in young patients: A case report

**DOI:** 10.1016/j.tcr.2026.101378

**Published:** 2026-05-21

**Authors:** Michael P. Kucharik, Nicholas Bostrom, Douglas L. Cerynik, Anjan R. Shah

**Affiliations:** a University of South Florida, 17 Davis Blvd, Tampa, FL, 33606, United States; b Stabiliz Orthopaedics, 574 Wharton Blvd, Exton, PA, 19341, United States; c Florida Orthopaedic Institute: 5901 E Fowler Ave, Temple Terrace, FL, 33617, United States

**Keywords:** Young displaced femoral neck fractures

## Abstract

Displaced femoral neck fractures in physiologically young patients remain a significant treatment challenge due to high complication rates associated with internal fixation, including osteonecrosis, non-union, and hardware failure.

This case report describes the successful use of a novel implant, the SimpliFix Hip System, in the surgical management of a 30-year-old female marathon runner who sustained a displaced transcervical femoral neck fracture. Following closed reduction on a fracture table, three partially threaded cannulated screws were inserted and cross-locked with headless cross screws using the SimpliFix system, which permits controlled compression and rotational stability while minimizing implant footprint. The construct was configured to impart rigidity at the calcar and controlled valgus compression to promote union.

Postoperatively, the patient was maintained toe-touch weightbearing for 10 weeks. Radiographs at routine follow-up demonstrated anatomic healing without evidence of implant failure or loss of reduction. At one year, the patient had resumed pre-injury activity levels, running 50 miles per week without pain or limitation. This case demonstrates the potential advantages of the SimpliFix system in young, active patients, particularly its capacity to resist femoral neck shortening, maintain reduction, and enhance biomechanical stability while preserving minimally invasive technique.

The positive clinical and radiographic outcome highlights the promise of this implant as an alternative to conventional compression screws or fixed-angle devices. Further studies are warranted to assess long-term outcomes and comparative efficacy.

## Introduction

While arthroplasty is a preferred for the treatment of displaced femoral neck fractures in the elderly, the optimal treatment for physiologically young patients remains a challenge for orthopaedic surgeons. Internal fixation of displaced femoral neck fractures have been associated with relatively high rates (20%–50%) of complications, including; osteonecrosis, non-union, hardware failure, and screw cut out [1], [2]. Complex biomechanical forces and tenuous intracapsular blood supply are the major hurdles in successful treatment. Despite significant technological advances among compression screws and fixed-angle dynamic implants, reported rates of osteonecrosis and non-union are as high as 45% and 30%, respectively [3], [4]. To mitigate these complications, an implant must resist femoral neck shortening, provide compression across the fracture, offer rotational stability, and limit its footprint. We present a case report of a 30-year-old female marathon runner who sustained a displaced femoral neck fracture and was subsequently treated with closed reduction and internal fixation using an innovative implant.

## Case presentation

**Fig. 1:**
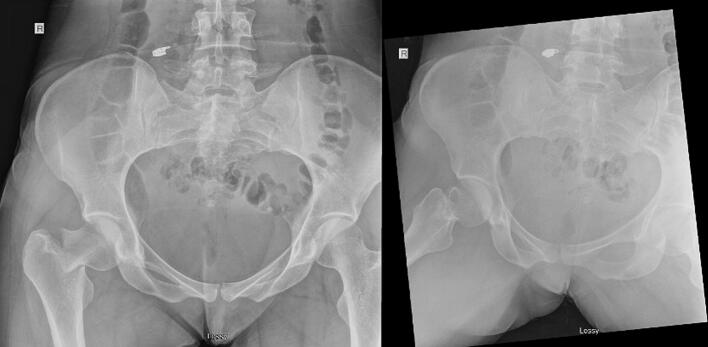
Anterior-posterior (AP) and lateral radiographs demonstrating a right displaced, transcervical femoral neck fracture.

A 30-year-old female sustained a right femoral neck fracture following a ground level fall. Given her reported history of antecedent right hip pain, she presumably had a pre-existing tension-sided stress fracture. The patient's medical history was remarkable for low vitamin D. The patient's social history included competitive marathon running, as she reported running on average 50 miles per week. Upon presentation, radiographs were remarkable for a right transcervical femoral neck fracture. Classified as a Pauwels II or Garden type III [5] (Fig. 1). No other associated injuries were identified. After a comprehensive discussion with the patient regarding possible treatment options, it was mutually decided to pursue operative fixation to restore her native anatomy.

The patient was subsequently brought to the operating room and placed on a fracture table in the supine position. The right hip was then closed reduced using gentle, sustained traction in-line with the femoral neck, with the hip flexed and externally rotated to untwist the spiral fibers of the hip capsule as previously described by Flynn [6]. The hip was then internally rotated, gradually extended and abducted to maintain reduction by tightening the spiral fibers of the capsule. Intraoperative fluoroscopy was used to confirm anatomic closed reduction.

After sterile preparation and draping, a 4 cm incision was made adjacent to the lateral aspect of the right hip. The fascia at this level was incised in line with the incision to expose the lateral femur. Three guide pins were then placed to transfix the femoral neck fracture, which were subsequently drilled, sized, and replaced with partially threaded 8.3-mm cannulated screws (SimpliFix Hip System, StabilizOrtho, Exton, PA). Using an aiming guide, the partially threaded cannulated screws were then transfixed with headless 3.7-mm SimpliFix Cross Screws. Unique to this system is the ability to independently cross-lock each cannulated screw within the slot at either the designated “0” or “3” position, representing the desired amount of compression/sliding.

**Fig. 2:**
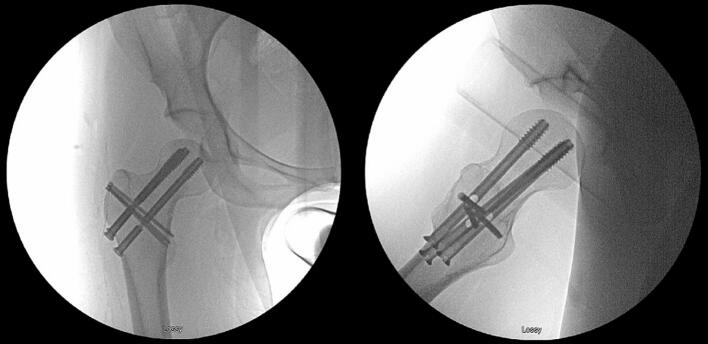
Intraoperative final AP and lateral radiographs following anatomic reduction and fixation of the displaced femoral neck fracture using the SimpliFix implant.

In this particular case, the surgeon wanted controlled compression of the fracture, encouraging a valgus vector over varus. The inferior screw was crossed at the “0” location, theoretically imparting more rigidity at the calcar region, and both the superior anterior and superior posterior screws were crossed at “3” allowing for controlled compression into valgus should the fracture need it for union. Intraoperative fluoroscopy confirmed that the implants were optimally positioned and that the fracture was stable to manipulation (Fig. 2).

**Fig. 3:**
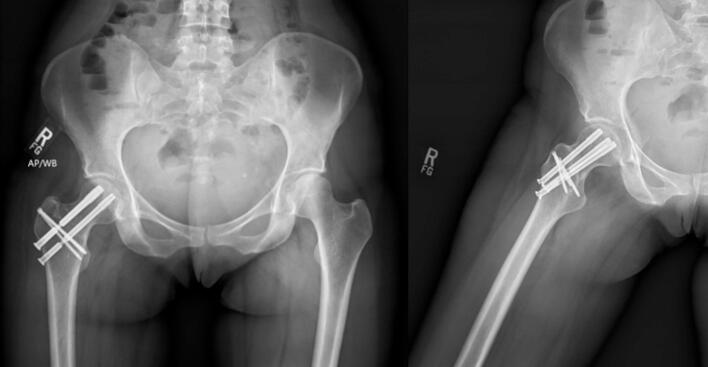
Postoperative AP and lateral radiographs from the patient's 10-week follow-up appointment demonstrating persistent anatomic reduction with no evidence of motion or radiolucency about the partially threaded screws across the fracture nor the upper screws that were inserted in the slotted holes.

**Fig. 4:**
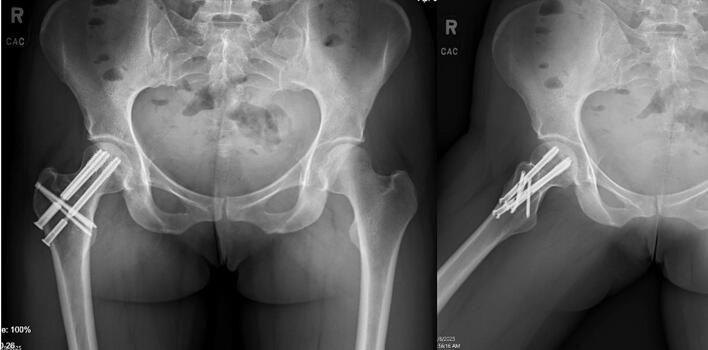
Postoperative AP and lateral radiographs from the patient's 1-year follow-up appointment demonstrating a successful radiographic outcome. The fracture has healed anatomically and there is no evidence of screw migration, cutout, or loosening.

Postoperatively, the patient was made toe-touch weightbearing about the operative extremity for 10 weeks. Interval radiographic and clinical examination at subsequent follow-up appointments confirmed anatomic healing of the fracture with full strength and pain-free uninhibited range-of-motion about the injured hip. Moreover, at her 1-year follow-up appointment, she reported that she had returned to her pre-injury function of running 50 miles per week (Fig. 3, Fig. 4).

## Discussion

Treating displaced femoral neck fractures in young patients presents a unique and demanding clinical challenge. Unlike geriatric patients, where arthroplasty is often the preferred solution for displaced fractures, preserving the native femoral head in younger individuals is critical due to their high functional demands. However, internal fixation in this population can be fraught with complications. The femoral head's vascular supply is highly susceptible to disruption during injury or surgical intervention, increasing the risk of avascular necrosis. Additionally, the orientation of the fracture itself requires consideration in choosing the correct fixation strategy [7], [8].

The optimal treatment strategy for displaced femoral neck fractures in young patients is a topic of debate. It is the authors opinion that first an anatomic reduction must be achieved, either closed or open. In the case of high-energy, vertical shear type fractures (i.e. Pauwels III) the preferred treatment remains a combination of inferior medial plating, screw and side plate, and **an** anti-rotation screw. In the case of displaced Pauwels I and II fractures, fixation constructs such as SimpliFix may be preferred as it has previously shown success in lowering complication and reoperations rates in Garden I & II fractures [9]. Even with theoretical lower complication rates, secondary surgery may occur. The limited footprint of the system has not resulted in bone loss or *peri* implant fractures, and in conversion procedures, primary arthroplasty implants have been successfully used. The femoral neck screws are easily exposed through the commonly used approaches for hip surgery. The crossing screw is reproducibly found at the greater trochanteric tip, and, if necessary, can be assisted by using either the targeting arm or disposable template offered in the system. This system's unique ability to limit shortening and control rotation, while maintaining a percutaneous approach warrants further investigation.

## Conclusion

This report highlights the use of a novel implant that offers a promising alternative to traditional compression screws or fixed-angle dynamic devices for managing displaced femoral neck fractures in young patients.

## CRediT authorship contribution statement

**Michael P. Kucharik:** Writing – review & editing, Writing – original draft, Methodology, Formal analysis, Data curation, Conceptualization. **Nicholas Bostrom:** Writing – review & editing, Writing – original draft, Investigation, Formal analysis, Data curation, Conceptualization. **Douglas L. Cerynik:** Writing – review & editing, Writing – original draft, Supervision, Project administration, Methodology, Investigation. **Anjan R. Shah:** Writing – review & editing, Writing – original draft, Supervision, Project administration, Investigation, Conceptualization.

## Declaration of competing interest

The authors declare the following financial interests/personal relationships which may be considered as potential competing interests:

Doug Cerynik, MD MBA – CEO and Director of StabilizOrtho

Anjan Shah, MD – Paid consultant for StabilizOrtho

Nicholas Bostrom, MD and Michael Kucharik, MD – No conflicts of financial interests/personal relationships which may be considered as potential competing interests.
